# Round the Hospitals

**Published:** 1916-10-28

**Authors:** 


### ROUND THE HOSPITALS.

Those nurses who have not yet received answers to their applications to register with the College of Nursing will understand the delay when they learn that a new circular has been issued with regard to the qualifications for registration, which are now more clearly defined. The applications from nurses who did not receive their training in any recognised school have consequently been held over, so that all may be included whose qualifications entitle them to membership. It will be noticed that for the purpose of admitting practising nurses to the Register, general civil hospitals and infirmaries to be recognised for training must have at least forty beds in daily occupation throughout the year. In the case of Poor-Law infirmaries they must contain not fewer than 250 beds; in both classes of institutions it is a condition that there is a resident medical or surgical officer, and that one course of nursing lectures is given annually, followed by examination for qualification. The new conditions are published in full on p. 75 of this week's issue.

---

The articles we have been publishing entitled "Patriotism and the Nurse-Training Schools" disclose a splendid record of war service on the part of the nursing staffs of our hospitals up and down the country. They have shown what has been done, since the outbreak of war, at twenty-two hospitals and eleven Poor-Law infirmaries. Where this record has not been procurable a chance examination reveals the same conditions, of staffs depleted for war service, existing at the majority of our great hospitals. At Aberdeen Royal Infirmary, for instance, the matron and assistant matron were mobilised on the outbreak of war, the former as principal matron of the 1st Scottish General Hospital. No trained nurses have been retained who wished to join the Naval or Military Service, with the consequence that the matron is left with only eight sisters and eighty probationers. Since August 1914 all the nurses, on completing their training, with the exception of six, immediately entered the Naval and Military Services. Yet the work of the hospital has to continue and does continue unimpaired, to the infinite credit of the matron, her staff, and all concerned. These conditions are but typical of those in every other hospital, a fact which the public probably does not sufficiently appreciate or comprehend.

---

Miss Mary Girdlestone, who is retiring from the office of matron of Crumpsall Infirmary after holding her post for twenty years, took advantage of the occasion of a presentation to her last week to speak of the changes and improvements which had taken place during her term of office. She rightly pointed out that improvements in the wards and the methods of nursing, though excellent indications of progress, would not by themselves produce good nurses. For this the right spirit and a high ideal of work and conduct were needed, and she hoped the Crumpsall nurses, under their new matron, a Crumpsall-trained nurse, would always keep this ideal before them, and would carry on the old traditions. She urged them always to hold together, to work together, and to remember that union is strength. The gift presented to Miss Girdlestone by past and present nurses was a writing bureau after a Hepplewhite design.

---

The excellent work done by the Local Ladies' Committees of the Territorial Force Nursing Service is too little realised. In addition to the duty of recruiting nurses under the regulations of this Service, these Committees have also rendered valuable assistance in supplementing the efforts of the working guilds—rolling bandages, covering splints, making tray-cloths, and washing and repairing various small articles. They have proved to be very active and of great assistance to the hospitals. In Newcastle-on-Tyne the Ladies' Committee of the 1st Northern General Hospital has undertaken the work of providing extra garments and other requirements for the wounded. In this branch of work they have had the co-operation of the ladies of the Royal Victoria Infirmary Ward Garment Guild, and together they have been able to render invaluable service for the wounded in the 1st Northern General Hospital. No less than 55,000 articles were provided during the year ending July 31, 1916, in addition to fruit, vegetables, cakes, sweets, flowers, tobacco, cigarettes, matches, notepaper, envelopes, books, cards, games, etc., while the funds show a satisfactory balance carried forward of £108 13s. 6d.

---

Many sisters must wish, when they see some patients go home, that these patients could still be in the hands of a trained nurse, or that they could, at all events, be followed up and some further help given them, even when there is no nursing treatment to be carried out. This system has been in vogue at St. Thomas's Hospital for some time. It is constantly being developed, and the work enlarged, so that both in-patients and out-patients may be followed up, and any help given them which they require. The work is carried out by the Lady Almoner's department. This department is not only responsible for finding out the home conditions of the patients, and arranging for visits to be paid by either Queen's or Ranyard nurses, but if the patient is not fit to do her housework, a "home help" is provided. When it is found that discharged in-patients contemplate returning to work which would probably only make them ill again, the Visiting Almoner tries to get other and suitable work for them. Another branch of this work is the provision of artificial teeth, spectacles, artificial limbs, and trusses. Through the Lady Almoner's department arrangements are made for patients to go to convalescent homes; the necessary clothes and an escort to the station are all provided. A complete record is kept of the history and the progress of tubercular patients who are attending the out-patient department; their homes are visited and suggestions made which will enable the patient to live under better conditions, and to be as little source of infection as possible to the other inmates of the house. The cost of this Almoner's department is considerable, but though it would probably be difficult to prove in figures, there is no doubt that its work not only saves a large sum of money to the hospital, but the surgeons', physicians', and nurses' work is made of lasting benefit. The Lady Almoner at St. Thomas's has eighteen workers under her, and she trains ladies for the work. One of her pupils has recently gone to Glasgow and another to Birmingham.

For College of Nursing New Conditions of Registration, see p. 75. For Matrons' and Sisters' Appointments, see p. 76.

